# Simulation and Experiment of Substation Grounding Resistance Measurement Based on Pseudorandom Signal

**DOI:** 10.3390/s22155777

**Published:** 2022-08-02

**Authors:** Qingming Duan, Bofeng Zou, Shanshan Li, Hu Zhao

**Affiliations:** College of Instrumentation & Electrical Engineering, Jilin University, Changchun 130012, China; zoubf21@mails.jlu.edu.cn (B.Z.); liss19@mails.jlu.edu.cn (S.L.); zhaohu20@mails.jlu.edu.cn (H.Z.)

**Keywords:** grounding resistance, pseudorandom signal, correlation function, system identification

## Abstract

The grounding resistance of a substation is an important parameter that should be designed within a reasonable range to prevent operational accidents from damaging electrical equipment due to overvoltage and ensure the safe operation of an electrical system. However, simply and accurately measuring the grounding resistance of a substation has been a difficult problem faced by engineers and technicians for a long time. This paper proposes a method of denoising by applying the m-sequence correlation identification technology to the measurement of substation resistance. We established a grounding resistance model of a grounding grid and used LabVIEW to simulate it. Based on system identification and correlation function theory, pseudorandom signals or sinusoidal signals were used as excitation signals. The output results of the system were compared when pseudorandom signals and sinusoidal signals were used as excitation signals. It was verified that the grounding resistance value measured by a pseudorandom signal was closer to the actual value, which met the design requirements. Laboratory test results verify that the method of calculating grounding resistance based on the correlation analysis method is feasible.

## 1. Introduction

Grounding resistance is the main technical index of a grounding system for power plants and substations. The effectiveness and safety of a grounding system are judged by grounding resistance. However, the measurement of grounding resistance is a complicated problem because it can be affected by many factors. It is not only related to the size, shape, and ground resistivity of the grounding device [1] but also affected by the surrounding electromagnetic field, metal substances in the soil, the uniformity of earth resistivity, the measurement method, and the electrode arrangement [2,3]. In practice, substation accidents are more common in the substation system environment. An investigation showed that China has had many direct or indirect accidents caused by grounding resistance not meeting requirements or excessive grounding resistance caused by aging grounding grids, accounting for approximately 30% of grid operation accidents [4]. Therefore, before each physical substation is put into operation, it is necessary to carry out systematic and scientific power grid testing, specifically to master the grounding resistance design of the grounding grid to meet the operational requirements of the substation [5]. Moreover, after a substation is put into operation, it is necessary to regularly check the grounding resistance parameters of the grounding grid of the substation and to adjust the substation system equipment in real time to meet the actual operation requirements. If a device is unqualified, it must be replaced or repaired to ensure the safe operation of electrical equipment [6].

The measurement methods currently studied mainly include the fall-of-potential method, the heterofrequency measurement method, the moment method (MM), and the finite element method (FEM). At present, the IEEE standards and China’s power industry standards are still based on the theory of the fall-of-potential method and its derivative methods for designing grounding resistance measurement devices. The fall-of-potential method is a basic method widely used in grounding impedance measurements [7]. The 0.618 method and the 29° method are simple applications of the fall-of-potential method. The fall-of-potential method needs to arrange a voltage electrode and a current electrode during measurement. However, there are mutual inductance interference phenomena between the leads of the voltage electrode and the current electrode. Dr. Jozsef Ladanyi introduced a method of measuring grounding resistance by the fall-of-potential method and its error in field measurement [3]. J. Ma analyzed the inductive coupling between the current lead and the voltage lead under a typical lead separation distance [8]. M. A. Salam measured the soil resistivity near the test site. Based on the fall-of-potential method to measure the grounding resistance near the substation, an empirical relationship between the grounding resistance and the distance between the grid and the current electrode was proposed [9]. The heterofrequency measurement method uses a unique hardware and software anti-interference method. Under the condition that the test current frequency is close to the system power frequency resistance, stable and reliable measurement results can be obtained [10]. References [11,12] proposed a theoretical analysis and measurement system for grounding impedance based on short current leads and swept frequency alternating current (AC) sources. The disadvantage of the heterofrequency measurement method is that it still needs to place two leads of voltage and current, and it is difficult to completely avoid the mutual inductance between the measuring leads. The MM [13] and the FEM [14,15] encounter problems when dealing with large-scale grounding grid problems, such as difficulties in subdivision, large amounts of calculation, and difficulties in fully considering various factors [16]. The main disadvantage is that when the size of the grounding grid is too large, the capacity of the computer is limited, especially when the ratio of the size of the grounding grid to the size of the grounding electrode is large. Pseudorandom signals are easy to generate and implement, and their statistical characteristics are similar to those of band-limited white noise. Within a certain frequency range, the power spectrum is almost uniformly distributed [17]. The autocorrelation function of a pseudorandom signal is a triangular pulse, which is similar to the delta (δ) function. The advantage of pseudorandom signals is strong anti-interference that can be easily separated from interference noise. As long as this does not affect the normal operation of the system, the impulse response of the system can be identified. This approach is mostly used in applications such as substation [17,18] and bioimpedance measurements [19].

With an m-sequence pseudorandom signal as the excitation signal and system identification principle as the theoretical basis, the correlation identification technique can not only collect the resistance information but also mitigate the impact of noise and natural potential [20,21]. The m-sequence exhibits desirable autocorrelation characteristics. As the period of m-sequence is extended and the time width decreases, the overall autocorrelation function approaches the response function of the system increasingly, thus achieving the purpose of resistance measurement. On the basis of in-depth research into the m-sequence correlation identification method and correct simulation calculation, this paper proposes a method of denoising by applying the m-sequence correlation identification technology to the measurement of substation resistance. According to this idea, the process of denoising correlation identification was simulated, and its outcomes were analyzed [22]. Compared with the methods of numerical calculation that involve complex processing, for example, segmenting areas and establishing boundary conditions, the pseudorandom-signal-based correlation identification method purposed to measure grounding resistance is more effective in improving the efficiency and accuracy of measurement.

The main purpose of this paper is to simulate the grounding resistance model of a grounding grid based on LabVIEW software [23]. The simulation used a pseudorandom signal or sinusoidal signal as the excitation signal. An excitation signal superimposed with Gaussian white noise was injected into the grounding resistance model to obtain a response signal superimposed with Gaussian white noise. Correlation operations were performed on the excitation signal and response signal, and then FFT was performed on the correlation function [24]. The grounding resistance value could be obtained through the power spectrum of the correlation function. The output results were compared when different excitation signals acted on the system. Finally, the practicability of the designed measuring device and the feasibility of the method used are verified by experiments.

## 2. Materials and Methods

In this section, we introduce the commonly used wiring methods when measuring grounding resistance. The concept of related functions is briefly described. The principle of system identification based on the cross-correlation method is analyzed. The theoretical knowledge of pseudorandom signals and their autocorrelation function are mentioned.

### 2.1. Grounding Resistance Measurement Method

**Figure 1:**
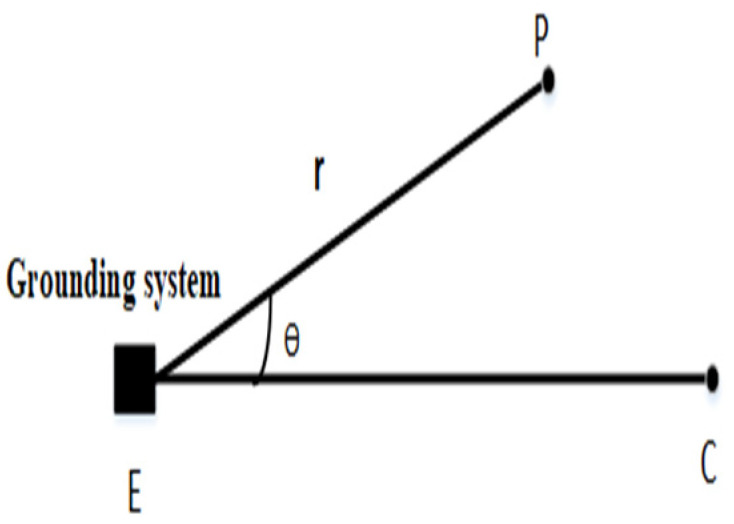
Commonly used arrangement when measuring grounding resistance using the fall-of-potential method.

Theoretically, the grounding resistance is equal to R = V/I, where V is the ground potential rise (GPR) of the grounding system relative to the remote soil, and I is the current injected into the grounding grid. Note that the injection current is assumed to return from infinity to the current electrode (return electrode). However, in reality, it is impossible to place the current electrode at infinity when injecting current into the grounding grid. Similarly, when measuring the GPR of a grounding system, it is impossible to place the voltage electrode at infinity [25]. In the measurement of the fall-of-potential method [26,27,28,29], the position of the voltage electrode needs to be continuously adjusted. The position distribution of the voltage electrode and the current electrode in the test is shown in Figure 1, which shows the commonly used arrangement when using the fall-of-potential method to measure the grounding resistance.

**Figure 2:**
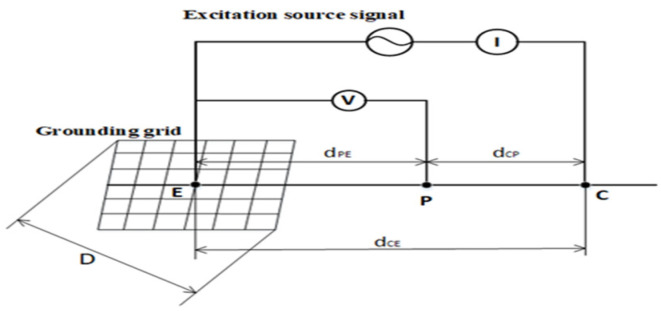
The voltage electrode and current electrode are located on the same side of the grounding grid. D is the diagonal length of the grounding grid.

In Figure 1, E is the ground electrode. P is the potential electrode. C is the current electrode. r is the distance between P and E. θ is the angle between P and C. When θ = 0°, this represents the 0.618 method derived from the fall-of-potential method. A schematic diagram of the arrangement of the voltage electrode and the current electrode is shown in Figure 2.

To facilitate the calculation, the grounding grid is equivalent to a hemisphere. According to the electromagnetic field calculation method, the voltage $U\left(d_{\mathrm{PE}}\right)$ at the voltage electrode can be obtained as

(1) $$U\left(d_{PE}\right)=\frac{\rho I}{2\pi d_{PE}}-\frac{\rho I}{2\pi (d_{CE}-d_{PE)}}$$

After Equation (1),  I is the current injected into the grounding grid, $U\left(d_{PE}\right)$ is the voltage between the two points P and E, ρ is the resistivity,$\;d_{PE}$ is the distance between the ground electrode and the voltage electrode, $d_{CE}$ is the distance between the ground electrode and the current electrode, and $d_{CP}$ is the distance between the voltage electrode and the current electrode. If R is the grounding resistance of the grounding grid, the voltage is

(2) $$U=U\left(0\right)=\mathrm{RI}-\frac{\rho I}{2\pi d_{CE}}$$

where U is the voltage at the reference point. Then, the potential difference is

(3) $$V=U-U\left(d_{\mathrm{PE}}\right)$$

(4) $$V=\mathrm{RI}-\frac{\rho I}{2\pi }\left(\frac{1}{d_{PE}}-\frac{1}{d_{CP}}+\frac{1}{d_{CE}}\right)$$

where V is the ground potential rise (GPR) of the grounding system.

Let $d_{PE}$ be a variable; the solution can be $d_{PE}=0.618d_{CE}$ [30,31]. That is, when the voltage electrode is located between the ground electrode and the current electrode and is $0.618d_{CE}$ away from the ground electrode, an accurate voltage value can be obtained. In fact, the grounding grid structure falls between the disk electrode and the ring electrode. Therefore, when the 0.618 method is used for grounding resistance detection, usually $d_{PE}=\left(4-5\right)D$ [32,33], $d_{PE}=\left(0.5-0.6\right)d_{\mathrm{CE}}$. If the size of the ground electrode is much smaller than $d_{PE}$ and $d_{CE}$, the above relationship is still valid. 

### 2.2. System Identification and Correlation Functions

For a signal $x\left(t\right)$ with a period of T, the average characteristic of its own correlation degree is called the autocorrelation function $R_{x}\left(\tau \right)$, which represents the average correlation degree between two different moments separated by τ, namely,

(5) $$R_{x}\left(\tau \right)=\underset{T\to \infty }{\lim}\frac{1}{T}\int_{0}^{T}x\left(t\right)x\left(t+\tau \right)\mathrm{dt}$$

If a linear system exists, the input signal is $x\left(t\right)$, the output signal is $y\left(t\right)$, and the input value $x\left(t\right)$ at time  t has an impact on the output value $y\left(t+\tau \right)$. The degree of this impact is the cross-correlation function $R_{\mathrm{xy}}\left(\tau \right)$ to describe, namely,

(6) $$R_{\mathrm{xy}}\left(\tau \right)=\underset{T\to \infty }{\lim}\frac{1}{T}\int_{0}^{T}x\left(t\right)y\left(t+\tau \right)\mathrm{dt}$$

**Figure 3:**
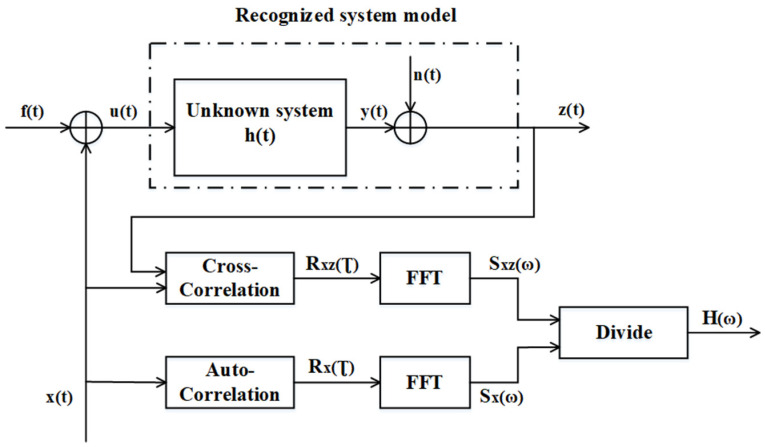
Principle of system identification based on the cross-correlation method.

The principle of using the cross-correlation method to identify the dynamic characteristics of the system is shown in Figure 3.

$R_{\mathrm{xz}}\left(\tau \right)$ is the cross-correlation function of the input signal $x\left(t\right)$ and the output signal $z\left(t\right)$, $R_{x}\left(\tau \right)$ is the autocorrelation function of the test signal $x\left(t\right)$, $S_{\mathrm{xz}}\left(\omega \right)$ is the power spectrum of the cross-correlation function $R_{\mathrm{xz}}\left(\tau \right)$, and $S_{x}\left(\omega \right)$ is the power spectrum of the autocorrelation function $R_{x}\left(\tau \right)$. The impulse response function is $h\left(t\right)$, all internal noises are modeled as noise $n\left(t\right)$ superimposed on the response signal $y\left(t\right)$, and the observed output signal $z\left(t\right)$ is

(7) $$z\left(t\right)=y\left(t\right)+n\left(t\right)$$

The test signal $x\left(t\right)$ is broadband noise, which is superimposed on the normal operating signal $f\left(t\right)$, and the signal input to the identified system is

(8) $$u\left(t\right)=x\left(t\right)+f\left(t\right)$$

where $u\left(t\right)$ is the sum of the signals $x\left(t\right)$ and $f\left(t\right)$. Generally, $f\left(t\right)$ and $x\left(t\right)$ are not related to each other.

For a physically achievable linear system, the input and output signals meet the following convolution relationship:

(9) $$y\left(t\right)=h\left(t\right)*u\left(t\right)=\int_{0}^{\infty }h\left(\alpha \right)u\left(t-\alpha \right)d\alpha$$

By performing cross-correlation processing on the test signal $x\left(t\right)$ and the observed output signal $z\left(t\right)$, we obtain

(10) $$R_{\mathrm{xz}}\left(\tau \right)=E\left\{x\left(-\tau \right)\left[y\left(t\right)+n\left(t\right)\right]\right\}$$

where $R_{\mathrm{xz}}\left(\tau \right)$ is the cross-correlation function of the input signal $x\left(t\right)$ and the output signal $z\left(t\right)$. Substituting (9) into (10) obtains

(11) $$R_{\mathrm{xz}}\left(\tau \right)=R_{\mathrm{xn}}\left(\tau \right)+\int_{0}^{\infty }h\left(\alpha \right)\left[R_{x}\left(-\alpha \right)+R_{\mathrm{xf}}\left(\tau -\alpha \right)\right]d\alpha$$

After Equation (11), $R_{\mathrm{xn}}\left(\tau \right)$ is the cross-correlation function of the input signal $x\left(t\right)$ and the noise $n\left(t\right)$.${\;R}_{\mathrm{xf}}\left(\tau -\alpha \right)$ is the cross-correlation function of the input signal $x\left(t\right)$ and the normal operating signal $f\left(t\right)$ at time $\left(\tau -\alpha \right)$. $R_{x}\left(-\alpha \right)$ is the autocorrelation function of the test signal $x\left(t\right)$ at time α. The test signal $x\left(t\right)$ is not related to the noise $n\left(t\right)$ and the production signal $f\left(t\right)$, so

(12) $$R_{\mathrm{xn}}\left(\tau \right)=R_{\mathrm{xf}}\left(\tau \right)=R_{\mathrm{xf}}\left(\tau -\alpha \right)=0$$

Bringing this solution into (11) obtains

(13) $$R_{\mathrm{xz}}\left(\tau \right)=h\left(\tau \right)*R_{x}\left(\tau \right)$$

where $R_{x}\left(\tau \right)$ is the autocorrelation function of the test signal $x\left(t\right)$ at time τ. The relationship expressed by (13) is called the Wiener–Hopf formula. Note that the existence of any signals other than $x\left(t\right)$ in the system does not affect the cross-correlation result of (13). Regardless of whether other signals are applied externally or generated inside the system, as long as they are not related to the test signal, the above conclusion is valid.

If the test signal $x\left(t\right)$ is white noise, then its autocorrelation function $R_{x}\left(\tau \right)$ is a δ function, and its power is set to K, which can be obtained by (13):

(14) $$h\left(t\right)=\frac{1}{K}R_{\mathrm{xz}}\left(t\right)$$

The impulse response function of $h\left(t\right)$ the system can be directly obtained from the cross-correlation function, and the frequency response function of the identified system can be obtained by the FFT of (14):

(15) $$H\left(\omega \right)=\frac{1}{K}S_{\mathrm{xz}}\left(\omega \right)$$

where $S_{\mathrm{xz}}\left(\omega \right)$ is the power spectrum of the cross-correlation function $R_{\mathrm{xz}}\left(\tau \right)$. $H\left(\omega \right)$ is the frequency response function. From the above analysis, we can see that when the input signal is random white noise, the system impulse response can be obtained by the correlation between the input signal and the output response. The amplitude of this impulse response becomes K times the original amplitude. The phase has not changed, and the shape is consistent with the real impulse response. Therefore, using white noise as the input signal, the system impulse response can be obtained through the cross-correlation method. However, in practice, white noise cannot be directly generated. Therefore, researchers often generate sequences with white noise properties to give them the correlation properties of random signals. In general, the m-sequence pseudorandom signal has periodicity, but as long as its period is much longer than the width of the autocorrelation function, it can be approximately considered to have the characteristics of white noise.

**Figure 4:**
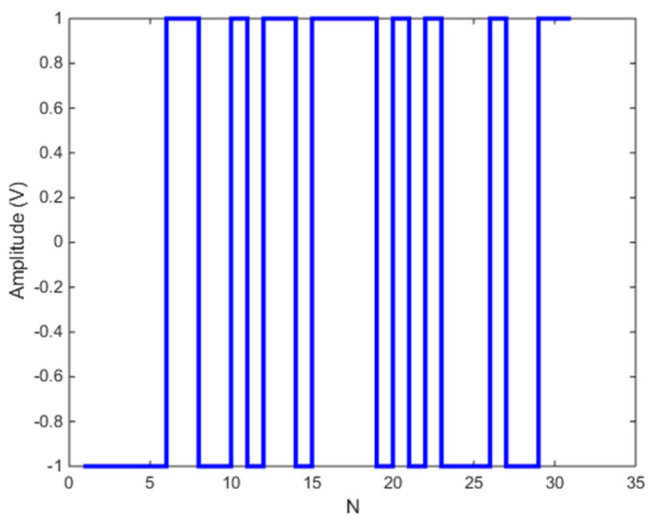
Five-level m-sequence pseudorandom signal.

The m-sequence is the earliest widely used pseudorandom sequence and has good randomness and balance. The m-sequence is called the maximum length shift register sequence (MLS), which is a positive- and negative-level signal. The n-stage shift register can be used to generate an m-sequence with length N = 2^n^ − 1. The m-sequence with an amplitude of ±1 generated by the 5-stage shift register is shown in Figure 4.

The autocorrelation function $R_{x}\left(\tau \right)$ of the bipolar m-sequence pseudorandom signal $x\left(t\right)$ with a level of ±a, a clock period of Δt and a period of T=NΔt is a triangular wave similar to the δ function. The autocorrelation function of the pseudorandom signal $x\left(t\right)$ is

(16) $$R_{x}\left(\tau \right)=\left\{\begin{array}{c} a^{2}\left[1-\frac{\left|\tau \right|}{\Delta t}\frac{N+1}{N}\right],\;\;\;\;\;-\Delta t\leq \tau \leq \Delta t\\ \;\;\;\;-\frac{a^{2}}{N},\;\;\;\;\;-\left|\Delta t\right|\leq \tau \leq \left|\left(N-1\right)\Delta t\right| \end{array}\right.$$

When N is large enough, in the interval of 0~NΔt, $R_{x}\left(\tau \right)$ can be approximated as a δ function [34].

The test signal parameters used for system identification are the clock period Δt, signal length N, repetition period q, and signal amplitude ±a. Only by correctly selecting each parameter can an accurate impulse response be obtained. The following is the selection of signal length N and signal amplitude ±a. When the clock period Δt is determined, the signal length N is selected to satisfy NΔt>T. The rule for selecting N is $N\Delta t=\left(1.25-1.5\right)T_{s}$. If the adjustment time $T_{s}$ of the system is unknown and the maximum time constant $T_{\max}$ of the system is known, the signal length N is selected as $N=\left(1.25-1.5\right)*\left(4-5\right)T_{\max}$. The amplitude a of the pseudorandom signal should be as large as possible without affecting the normal operation of the system to improve the signal-to-noise ratio.

If $x\left(t\right)$ is a sinusoidal function $s\left(t\right)$ superimposed with uncorrelated noise $n\left(t\right)$, then

(17) $$x\left(t\right)=s\left(t\right)+n\left(t\right)=\mathrm{Asin}\left(\omega_{0}t+\varphi \right)+n\left(t\right)$$

In the formula, A is the signal amplitude, $\omega_{0}$ is the signal angular frequency, φ is the initial phase angle of the signal, and its autocorrelation function is

(18) $$R_{x}\left(\tau \right)=R_{s}\left(\tau \right)+R_{n}\left(\tau \right)=\underset{T\to \infty }{\lim}\frac{1}{2T}\int_{-T}^{T}\frac{-A^{2}}{2}\left[\cos\left(2\omega_{0}t-\omega_{0}\tau +2\varphi \right)-\cos\left(\omega_{0}\tau \right)\right]\mathrm{dt}+R_{n}\left(\tau \right)$$

where $R_{s}\left(\tau \right)$ is the autocorrelation function of the sinusoidal function $s\left(t\right)$. ${\;R}_{n}\left(\tau \right)$ is the autocorrelation function of the noise $n\left(t\right)$. The long-term integration result of the first cosine function in the integrand is zero, and the second term is not a function of time t, so

(19) $$R_{x}\left(\tau \right)=\frac{A^{2}}{2}\cos\left(\omega_{0}\tau \right)+R_{n}\left(\tau \right)$$

If $n\left(t\right)$ is broadband noise, $R_{n}\left(\tau \right)$ is concentrated around τ=0. When τ is large, the amplitude A and frequency f of the signal $s\left(t\right)$ can be measured by $R_{x}\left(\tau \right)$. In this way, after autocorrelation processing, the amplitude and frequency of the sinusoidal signal are extracted from the noise.

## 3. Design and Result Analysis

In this section, we built a pure resistance model and reasonably selected simulation parameters. The output results of the system when the m-sequence pseudorandom signal and sinusoidal signal were used as excitation signals were compared. The simulation results of grounding resistance when the signal was superimposed with or without noise were analyzed. Finally, laboratory test verify that is feasible.

### 3.1. Simulation Model Design and Parameter Design

**Figure 5:**
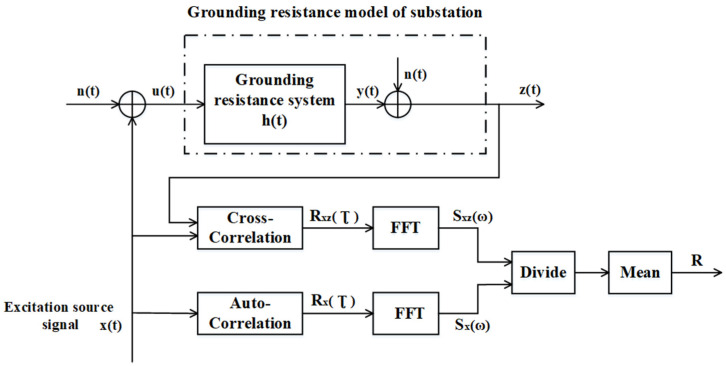
Grounding grid simulation model.

Figure 5 shows the grounding grid simulation model. An m-sequence pseudorandom signal or sinusoidal signal is used as the excitation signal $x\left(t\right)$ and injected into the grounding grid. The grounding resistance of the grounding grid is set to $h\left(t\right)$ to obtain the response signal $y\left(t\right)$. The response signal is superimposed with external interference noise $n\left(t\right)$ to obtain the output signal $z\left(t\right)$. The autocorrelation function of the excitation signal and the cross-correlation function of the excitation signal and the output signal are calculated. FFT is performed to obtain the power spectrum of the autocorrelation function and the cross-correlation function. The cross-correlation power spectrum is divided by the autocorrelation power spectrum to obtain the grounding resistance value, namely,

(20) R=SxzωSxω

where $S_{\mathrm{xz}}\left(\omega \right)$ is the power spectrum of the cross-correlation function $R_{\mathrm{xz}}\left(\tau \right)$, and $S_{x}\left(\omega \right)$ is the power spectrum of the autocorrelation function $R_{x}\left(\tau \right)$. R is the grounding resistance.

Then, take the average and calculate the final result, considering that in actual measurements, there is inevitable noise when using analog-to-digital conversion (ADC) to collect excitation signals and response signals. Therefore, in the simulation process, an interference noise signal $n\left(t\right)$ is also superimposed on the input signal $\;x\left(t\right)$. The interference noise signals used in the simulation are all Gaussian white noise signals.

In the simulation model, the sampling frequency of the excitation signal $x\left(t\right)$ was $F_{s}=1\;\mathrm{kHz}$, the number of sampling points $N_{0}$ of the signal was 1000, and the frequency resolution was $F=\frac{F_{s}}{N_{0}}=1$. The number of FFT sampling points $N\geq \frac{F_{s}}{F}$ and N was usually an integer power of 2, so $N=2^{10}=1024$ was selected in the simulation experiment. The standard deviation of the superimposed Gaussian white noise was 0.5. In practice, the noise superimposed by the collected signal is not overly large to better verify the characteristics of pseudorandom signals that are easy to separate from noise. We chose a larger intensity of noise.

### 3.2. Comparative Analysis of Sinusoidal Signal and Pseudorandom Signal

The main LabVIEW program was mainly composed of an excitation signal generation module, a signal superimposed noise module, an autocorrelation and cross-correlation signal module, an FFT module, and a result calculation module. First, set the standard value of resistance R1; second, inject the m-sequence pseudorandom signal or sinusoidal signal superimposed with noise into the pure resistance model as the excitation signal to obtain the current signal and voltage signal at both ends of the resistance. Then, the data were processed based on the correlation identification method to get the measurement result R2. By comparing the values of R1 and R2, the accuracy of R2 was verified.

**Figure 6:**
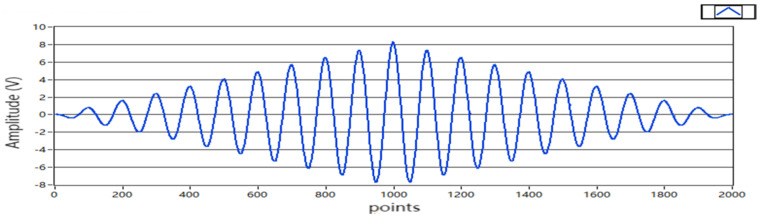
Sine signal autocorrelation function.

**Figure 7:**
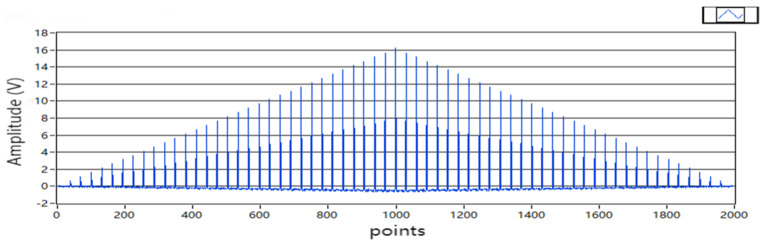
Pseudorandom autocorrelation function.

The amplitude of the sinusoidal signal was A=4, and the frequency was 10 Hz; the simulation waveform is shown in Figure 6. In the simulation experiment, the number of signal sampling points was selected to be 1000. If the signal is periodic, then its autocorrelation function is also periodic. The autocorrelation function of the sine signal is the cosine function of the energy attenuation. When τ=1000, Rx1000=A22=8, which conforms to the theoretical value. If the number of pseudorandom signal levels was n = 5, the signal length was N = 2^n^ − 1 = 31, and the amplitude was a=4. The simulation waveform is shown in Figure 7. When τ=1000, $R_{x}\left(1000\right)=a^{2}=16$. The other parameters of the two excitation signals and other parameters of the program were the same.

**Figure 8:**
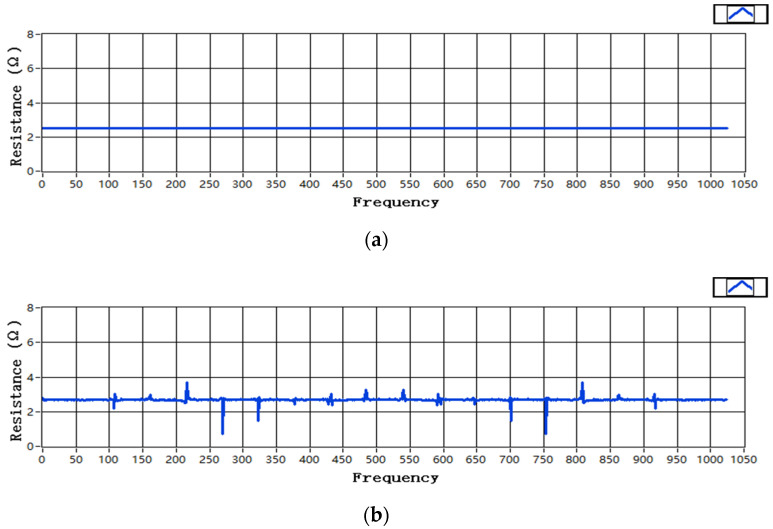
Curve of grounding resistance simulation results: (**a**) curve of grounding resistance without interference noise; (**b**) curve of grounding resistance with superimposed interference noise.

Figure 8 shows a curve diagram of the grounding resistance simulation results. Figure 8a shows that the resistance is a constant value when there is no interference noise, and the curve is a straight line. Figure 8b is the resistance curve after the interference noise is superimposed. Because of the presence of noise interference, the simulation result curve has a spike, and the final result can be approximated to the actual value by averaging.

**Figure 9:**
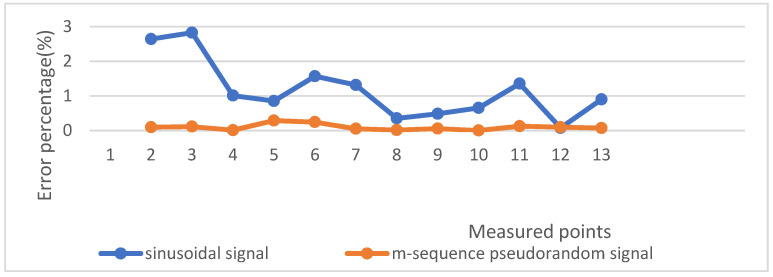
Percentage error curve of the sine signal and pseudorandom signal measuring resistance.

The grounding resistance in the substation grounding grid is usually less than 4 Ω, so a resistance value between 0–10 Ω was randomly selected for simulation. Table 1 shows the randomly selected resistance values and the grounding resistance values obtained by the simulation of the two excitation signals and their error percentages. In order to avoid stochastic uncertainty, we measured three groups of data for each measured point and calculated the average value of them. Then the error percentage between the average value and the standard value was calculated. Figure 9 shows the error percentage curve.

It can be seen in the above figures that when a sinusoidal signal is used as an excitation signal, the grounding resistance value obtained differs greatly from the actual value. Moreover, the data vary widely and are extremely unstable. The error percentage can reach 2.83%. The pseudorandom signal can be well separated from the signal. The grounding resistance value obtained by the simulation measurement is close to the standard resistance value, and the error percentage is also very small, basically below 0.29%, which is allowed in the actual device design. The grounding resistance measurement result is stable. Moreover, this result is obtained when the noise is relatively large. In practice, the superimposed noise of the signal is relatively small, which verifies that the pseudorandom signal as the excitation signal can identify the system well. There will be much interference in the surrounding environment of the substation, including harmonic interference and Gaussian interference. The pseudorandom signal is easy to separate from the noise, which shows its strong anti-interference ability.

In addition, the pseudorandom signal is a binary signal, which can be realized by turning off the switch. However, if a sinusoidal signal is used as the power supply current of the measuring device, its power supply voltage is usually not very low. The distortion will increase as the output amplitude increases. The greater the distortion is, the more inaccurate the measurement results are. This proves that pseudorandom signals can be more easily generated and accurately used for the measurement of substation grounding resistance.

### 3.3. Laboratory Test

**Figure 10:**
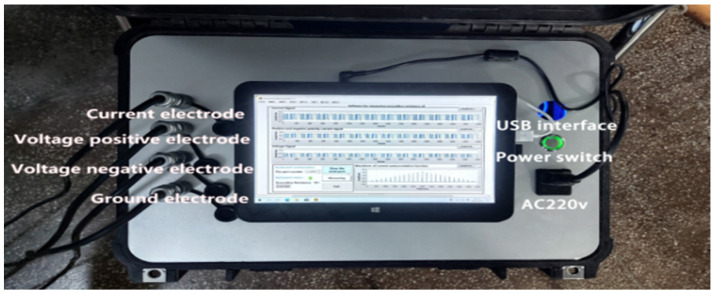
Schematic diagram of the grounding resistance measuring device and its connection: Measuring instrument.

According to the method proposed, a device was designed for the purpose of measuring the grounding resistance. The device consists of an m-sequence pseudorandom signal generation module, a solid-state relay module, a current sensor module, an ADC acquisition module, a serial communication module, and an upper computer LabVIEW module. STM32 single-chip microcomputer (STMicroelectronics, Geneva, Switzerland) is intended to generate the m-sequence pseudorandom signal treated as an excitation signal. The solid-state relay is equivalent to a non-contact switch purposed to separate the control end from the load end. After capturing the excitation signal, the current sensor converts it into a voltage signal, which is conducive to processing the signal for the single-chip microcomputer. As the core of the ADC acquisition circuit, the ADS1256 chip achieves the conversion between analog signals and digital signals. The upper computer LabVIEW module is responsible mainly for autocorrelation and cross-correlation operations, FFT calculation, and the display of results. A grounding resistance model was constructed in the laboratory, with the connection diagram shown in Figure 10.

**Figure 11:**
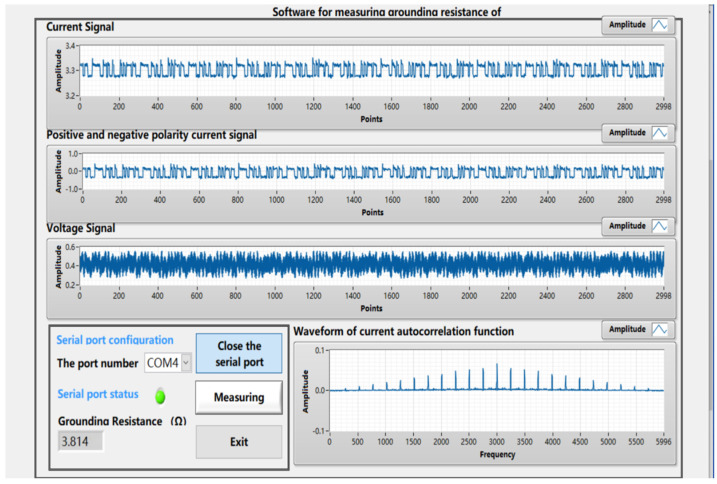
The LabVIEW interface displayed the signal waveforms and the measurement value of the grounding resistance.

In the model, the potential electrode, the current electrode, and the ground electrode were connected, with the power switched on for testing. The upper computer LabVIEW was used to display the m-sequence pseudorandom signal waveform, the voltage signal waveform, and the grounding resistance value, as shown in Figure 11. We conducted test on different resistance values, with the measurement results and their errors listed in Table 2.

It can be seen from the above figures and table that the error between the measurement value and the actual value is less than 4%, which falls within the allowable range. In the meantime, it was verified that the method of calculating the grounding resistance based on the correlation analysis method is feasible. In addition, the method demonstrates high precision and robustness to interference.

### 3.4. Discussion

Compared with numerical calculation methods that require complex processing procedures such as dividing areas and establishing boundary conditions, the correlation identification method based on pseudorandom signal is used to measure the grounding resistance to improve efficiency. In this section, a pure resistance model is built. Specifically, the sampling frequency $F_{s}$ of the excitation signal $x\left(t\right)$ and the number of sampling points $N_{0}$ of the signal in the simulation model were 1 kHz and 1000, respectively. In this paper, the superimposed noise is Gaussian white noise with a standard deviation of 0.5. Thus, the sampling frequency $F_{s}$ of 1 KHz will not be affected by the frequency of 50 Hz. The output results of the system were compared when pseudorandom signals and sinusoidal signals were used as excitation signals. The pseudorandom signal can be well separated from the signal. The grounding resistance value obtained by the simulation measurement is close to the standard resistance value. It verifies that the pseudorandom signal as the excitation signal can well identify the system and the laboratory test results verify that the method of calculating grounding resistance based on the correlation analysis method is feasible. Additionally, there is much interference in the surrounding environment of the substation, including harmonic interference and Gaussian interference. The pseudorandom signal is easy to separate from the noise, suggesting its strong anti-interference ability. 

## 4. Conclusions

This paper proposed a simulation experiment on the grounding resistance of the substation grounding grid. First, we analyzed the layout of the lead wires when measuring the grounding resistance of the substation. Then, based on the system identification and correlation function theory, a pseudorandom signal was used as the excitation signal, and autocorrelation and cross-correlation operations were performed. Then, FFT was carried out on the correlation function to measure the grounding resistance value in the simulation model. Finally, a comparative analysis with the sinusoidal signal was carried out, which verified that the pseudorandom signal was easy to separate from the noise interference signal and reflected its strong anti-interference characteristics. 

Through this simulation experiment, we carried out the design of a substation grounding resistance measurement device. Based on the theoretical methods used in the simulation experiment, considering various interferences around the substation, the hardware circuit was rationally designed. These circuits included signal acquisition circuits, power amplifier circuits, and signal conditioning circuits. Then, the algorithm used in the simulation experiment was used for data processing. Finally, a practical grounding resistance measurement device could be obtained.

In the actual measurement, to avoid the coupling problem between the current lead and the voltage lead, the short arrangement and accurate position of the lead as far as possible are required. Moreover, when arranging the electrode, technicians should avoid the underground metal and other places where electromagnetic interference will occur. Finally, high precision ADC is needed during the design of a grounding resistance measuring instrument. It can be used to realize accurate collection and conversion of the current signal and the voltage signal, thus increasing the accuracy of grounding resistance measurement.

## Figures and Tables

**Table 1:** Standard resistance values and grounding resistance values measured by two excitation source signals.

Standard Resistance Value (Ω)	Resistance Value Measured by Sinusoidal Signal (Ω)				Error Percentage (%)	Resistance Value Measured by M-Sequence Pseudorandom Signal (Ω)				Error Percentage (%)
	Group 1	Group 2	Group 3	Average Value		Group 1	Group 2	Group 3	Average Value	
0.656	0.67	0.67	0.68	0.67	2.64	0.67	0.65	0.65	0.66	0.10
1.698	1.64	1.66	1.65	1.65	2.83	1.69	1.7	1.71	1.7	0.12
2.273	1.97	2.45	2.33	2.25	1.01	2.27	2.27	2.28	2.27	0.01
3.547	3.48	3.6	3.47	3.52	0.86	3.53	3.54	3.54	3.54	0.29
4.588	4.67	4.66	4.65	4.66	1.57	4.57	4.59	4.57	4.58	0.25
4.754	4.94	4.82	4.69	4.82	1.32	4.78	4.74	4.75	4.76	0.06
5.341	5.32	5.37	5.39	5.36	0.36	5.34	5.34	5.34	5.34	0.02
6.217	6.21	6.16	6.19	6.19	0.49	6.21	6.22	6.21	6.21	0.06
6.543	6.44	6.46	6.6	6.5	0.66	6.52	6.56	6.55	6.54	0.01
8.421	8.05	8.37	8.5	8.31	1.36	8.4	8.41	8.42	8.41	0.13
9.234	9.43	9.11	9.14	9.23	0.08	9.24	9.25	9.24	9.24	0.10
10.568	10.65	10.65	10.69	10.66	0.90	10.55	10.56	10.57	10.56	0.08

**Table 2:** The grounding resistance measurement value and the error between the measurement value and the actual value.

The Actual Resistance Value (Ω)	The Measurement Resistance Value (Ω)	Error Percentage (%)
0.3	0.306	2.00
0.5	0.513	2.60
1.0	1.013	1.30
1.5	1.442	3.87
1.8	1.765	1.94
2	1.938	3.10
2.3	2.242	2.52
2.5	2.476	0.96

## References

[1] Pratama R.A., Hermawan, Facta M. Analysis of Grounding System in 150 kV Kudus Substation. Proceedings of the 2018 5th International Conference on Information Technology, Computer, and Electrical Engineering (ICITACEE).

[2] Unde M.G., Kushare B.E. Impact of seasonal variation of soil resistivity on safety of substation grounding system. Proceedings of the Fifth International Conference on Advances in Recent Technologies in Communication and Computing (ARTCom 2013).

[3] Ladanyi J., Smohai B. Influence of auxiliary electrode arrangements on earth resistance measurement using the Fall-of-Potential method. Proceedings of the 2013 4th International Youth Conference on Energy (IYCE).

[4] Li Y. (2017). Research on Short-Distance Measurement Method of Grounding Resistance of Large-Scale Substation Grounding Grid. Master’s Thesis.

[5] Phayomhom A., Sirisumrannukul S., Kasirawat T., Puttarach A. Safety design planning of ground grid for outdoor substations in MEA’s power distribution system. Proceedings of the ECTI-CON2010.

[6] Colak I., Garip I., Issi F. An application of maintaining constant grounding resistance of renewable energy sources by using a dspic. Proceedings of the 2014 Ninth International Conference on Ecological Vehicles and Renewable Energies (EVER).

[7] Nassereddine M., Rizk J., Nagrial M., Hellany A. Substation earth grid measurement using the fall of potential method (FOP) for a limited test area. Proceedings of the 2014 Australasian Universities Power Engineering Conference (AUPEC).

[8] Ma J., Dawalibi F.P. Effects of inductive coupling between leads in ground impedance measurements using the fall-of-potential method. Proceedings of the IEEE T&D Conference.

[9] Salam M.A., Noh M. Measurement of grounding resistance with square grid and rods near substations. Proceedings of the IEEE EPEC.

[10] Liu Y. (2019). Measurement method and application analysis of substation grounding resistance. J. Pop. Electric..

[11] Zhang B., Zeng R., He J., Sun W., Yao J., Su Q., Han S.W. Novel measurement system for grounding impedance of substations and power plants. Proceedings of the International Conference on PowerCon 2000.

[12] Lima A.B., Paulino J.O.S., Boaventura W.C., Guimarães M.F. Transient ground impedance measurement using a very short current lead. Proceedings of the 2013 International Symposium on Lightning Protection (XII SIPDA).

[13] Vieira P.H., Moura R.A., Schroeder M.A., Lima A.C. (2020). Symmetry exploitation to reduce impedance evaluations in grounding grids. Int. J. Electr. Power Energy Syst..

[14] Guemes J.A., Hernando F.E. (2004). Method for calculating the ground resistance of grounding grids using FEM. IEEE Trans. Power Deliv..

[15] Hong T.P., Van Q.D., Viet T.V. Grounding resistance calculation using FEM and reduced scale model. Proceedings of the IEEE CEIDP.

[16] Ma J., Dawalibi F.P. (2002). Analysis of grounding systems in soils with finite volumes of different resistivities. IEEE Trans. Power Deliv..

[17] Li F., Li Z., Li H., Yang Z., Gao P., Zhou W. Application of pseudo random correlation identification method based on LabVIEW in electromagnetic prospecting. Proceedings of the 37th CCC.

[18] Liu Y., Dong H., Liu X., He Z. (2010). A preliminary study on the application of m-sequence in electrical exploration. J. Eng. Geophys..

[19] Gao Y., Tang R., Liang J., Zhao T., Lu B., Jiang W. (2012). Identification of transmission characteristics of airborne noise in car based on m pseudo-random sequence. J. Jilin Univ. Eng. Technol. Ed..

[20] Song X., Wang X., Dong Z., Zhao X., Feng X. (2018). Analysis of Pseudo-Random Sequence Correlation Identification Parameters and Anti-Noise Performance. Energies.

[21] Cheng L. (2015). Research on Suppression of Controllable Source Electromagnetic Sounding Interference Based on M-Sequence Correlation Identification. Master’s Thesis.

[22] Liu J. (2010). Research on Correlation Identification of M-Sequence and Its Application in Induced Polarization Method and Denoising Analysis. Master’s Thesis.

[23] Wang H. (2013). Research and Implementation of Frequency Domain Correlation Identification Based on LabVIEW. Master’s Thesis.

[24] Zhi Q., Deng X., Wu J., Chen X., Zhang J., Wang X., Yang Y. (2018). Research on the anti-jamming mechanism of electromagnetic method correlation identification and numerical simulation. J. Prog. Geophys..

[25] Ma J., Dawalibi F.P. (2002). Extended analysis of ground impedance measurement using the fall-of-potential method. IEEE Trans. Power Deliv..

[26] Korasli C. (2005). Ground resistance measurement with alternative fall-of-potential method. IEEE Trans. Power Deliv..

[27] Colella P., Pons E., Tommasini R., Silvestre M.L.D., Sanseverino E.R., Zizzo G. (2019). Fall of Potential Measurement of the Earth Resistance in Urban Environments: Accuracy Evaluation. IEEE Trans. Ind. Appl..

[28] Dimcev V., Handjiski B., Sekerinska R. Alternative fall-of-potential method for grounding grids impedance measurements and inductive coupling between leads. Proceedings of the 2003 IEEE International Symposium on Electromagnetic Compatibility, EMC ’03..

[29] Dimcev V., Handjiski B., Vrangalov P., Sekerinska R. Impedance measurement of grounding systems with alternative fall-of-potential method. Proceedings of the Conference Record of the 2000 IEEE Industry Applications Conference, Thirty-Fifth IAS Annual Meeting and World Conference on Industrial Applications of Electrical Energy (Cat. No.00CH37129).

[30] Alcantara F.R. Simulation of Measurements of Resistances of Grounding Systems by a Simple Hemispheric Model. Proceedings of the 2019 IEEE XXVI International Conference on Electronics, Electrical Engineering and Computing (INTERCON).

[31] Alcantara F.R. An Approximated Procedure to Find the Correct Measurement Point in the Fall-of-Potential Method. Proceedings of the 2018 IEEE PES Transmission & Distribution Conference and Exhibition—Latin America (T&D-LA).

[32] Yu Z. (2018). The application research and improvement of the fall of potential method in the grounding impedance test of the grounding grid. J. Eng. Constr. Des..

[33] Tan H., Wu C., Zhang J. (2020). Research on grounding impedance test of substation grounding grid. J. Guangxi Acad. Sci..

[34] Li B.-N. (1987). Pseudo-Random Signal and Related Identification. http://book.sciencereading.cn/shop/book/Booksimple/onlineRead.do?id=BBB2FA6CD52C648C3B9551D48E541CE55000&readMark=1.

